# Production of functionalized polyhydroxyalkanoates by genetically modified *Methylobacterium extorquens *strains

**DOI:** 10.1186/1475-2859-9-70

**Published:** 2010-09-16

**Authors:** Philipp Höfer, Young J Choi, Michael J Osborne, Carlos B Miguez, Patrick Vermette, Denis Groleau

**Affiliations:** 1 Microbial and Enzymatic Technology Group, Bioprocess Centre, Biotechnology Research Institute, National Research Council Canada, 6100 Royalmount Avenue, Montréal, Québec, H4P 2R2, Canada; 2 Laboratoire de Bioingénierie et de Biophysique de l'Université de Sherbrooke, Department of Chemical and Biotechnological Engineering, Université de Sherbrooke, 2500 Boulevard de l'Université, Sherbrooke, Québec, J1K 2R1, Canada; 3 Biophysics and Nuclear Magnetic Resonance, Institute for Research in Immunology and Cancer, Université de Montréal, 2900 Boulevard Édouard-Montpetit, Montréal, Québec, H3T 1J4, Canada; 4 Current Address: Oswaldo Cruz Foundation (FIOCRUZ), Bio-Manguinhos, Viral Vaccine Program, Avenida Brasil 4365, 21045-900 Rio de Janeiro/RJ, Brazil

## Abstract

**Background:**

Methylotrophic (methanol-utilizing) bacteria offer great potential as cell factories in the production of numerous products from biomass-derived methanol. Bio-methanol is essentially a non-food substrate, an advantage over sugar-utilizing cell factories. Low-value products as well as fine chemicals and advanced materials are envisageable from methanol. For example, several methylotrophic bacteria, including *Methylobacterium extorquens*, can produce large quantities of the biodegradable polyester polyhydroxybutyric acid (PHB), the best known polyhydroxyalkanoate (PHA). With the purpose of producing second-generation PHAs with increased value, we have explored the feasibility of using *M. extorquens *for producing functionalized PHAs containing C-C double bonds, thus, making them amenable to future chemical/biochemical modifications for high value applications.

**Results:**

Our proprietary *M. extorquens *ATCC 55366 was found unable to yield functionalized PHAs when fed methanol and selected unsaturated carboxylic acids as secondary substrates. However, cloning of either the *phaC1 *or the *phaC2 *gene from *P. fluorescens *GK13, using an inducible and regulated expression system based on cumate as inducer (the cumate switch), yielded recombinant *M. extorquens *strains capable of incorporating modest quantities of C-C double bonds into PHA, starting from either C6= and/or C8=. The two recombinant strains gave poor results with C11=. The strain containing the *phaC2 *gene was better at using C8= and at incorporating C-C double bonds into PHA. Solvent fractioning indicated that the produced polymers were PHA blends that consequently originated from independent actions of the native and the recombinant PHA synthases.

**Conclusions:**

This work constitutes an example of metabolic engineering applied to the construction of a methanol-utilizing bacterium capable of producing functionalized PHAs containing C-C double bonds. In this regard, the PhaC2 synthase appeared superior to the PhaC1 synthase at utilizing C8= as source of C-C double bonds and at incorporating C-C double bonds into PHA from either C6= or C8=. The *M. ex-phaC2 *strain is, therefore, a promising biocatalyst for generating advanced (functionalized) PHAs for future high value applications in various fields.

## Background

Polyhydroxyalkanoates with functional groups (functionalized PHAs) have attracted increasing attention in the last ten years or so [1-9]. It has been generally recognized that important needs and markets exist for various types of functionalized PHAs as advanced materials in areas, such as tissue engineering [2,10,11], biocomposites [12], various medical applications [5,13], and polymers with tunable properties [6], only to name a few.

Although many types of functionalized PHAs have been described in the literature, generally speaking, the quantities produced have been very modest and barely sufficient to obtain a basic characterization of the physico-chemical properties of the new biomaterials [8]. Several reasons explain the situation and these include high toxicity of many of the key substrates, low accumulation of the desired PHAs and lack of commitment to developing more efficient, and productive fermentation processes.

A commonly applied route for obtaining polyhydroxyalkanoates with desirable functionalities is to produce PHAs with terminal double bonds followed by chemical modification steps. Carbon double bonds are comparatively inert but can be easily transformed into reactive functional groups under mild reaction conditions. Following this approach, polyhydroxyalkanoates with carboxyl, hydroxyl, epoxy and halogenic groups have been produced ([14] and references therein). *Pseudomonas *species have been most widely used for producing polyhydroxyalkanoates with double bonds in their side chains since they generate functionalized PHAs at comparably high rates of productivity. The incorporation of functional groups in PHAs by pseudomonads occurs by oxidizing functionality-related substrates via the *β*-oxidation pathway. The most commonly used substrate to obtain unsaturated polymeric side chains has been 10-undecenoic acid (C11=), likely owing to its superior availability compared to other alkenoic acids. The *β*-oxidation cycle is accompanied by a partial C2 reduction so that unsaturated monomers resulting from C11= exhibit a length of nine and, respectively, seven carbon atoms. The production of purely unsaturated polyhydroxyalkanoates was reported [15]; however, since chemical modification reactions do not require a double bond in every side chain and saturated substrates are cheaper, sodium alkanoate (C8) or nonanoate (C9) were often co-fed [16-18]. Using continuous culture mode, it was possible to produce structurally tailored poly(3-hydroxyalkanoate-*co*-3-hydroxyalkenoate)s with defined monomeric compositions [19]. Most functionalized PHAs that have been produced to date belong to the family of medium-chain-length (MCL, C ≥ 6) polyhydroxyalkanoates, in which all monomers have six carbon atoms or more. These PHAs are characterized by rubber-like mechanical properties with low melting temperatures (< 100°C). The combination of medium-chain-length with short-chain-length (SCL, C ≤ 5) monomers to SCL/MCL-PHAs resulted in copolymers with improved thermo-mechanical properties, however, without functionalities [20-23].

In this study, we present data showing that some newly developed recombinant *Methylobacterium extorquens *strains can deliver functionalized PHAs containing C-C double bonds when fed unsaturated fatty acids. These PHAs comprised short-chainlength (SCL, C ≤ 5) and medium-chain-length (MCL, 6 ≤ C ≤ 8) monomers and belong to a novel class of PHAs that will likely gain much attention due to their potential for high value applications in various fields including tissue engineering. One reason for choosing a methylotrophic microorganism for such purpose was that an important portion of the production process would use methanol, a simple, inexpensive, very abundant, and non-food substrate [24-26]. Another reason was that the project would contribute to establishing *M. extorquens *as a new cell factory, as strongly advocated in a recent review [27].

## Materials and methods

### Microorganisms

The proprietary, wildtype strain *Methylobacterium extorquens *ATCC 55366 was used throughout this study [24]. Fresh plates were prepared from vials stored at -80°C. Pre-inocula or inocula were prepared from plates stored at 4°C. The recombinant *M. extorquens *strains were similarly stored except that medium contained tetracycline at 15 mg/L to maintain selective pressure.

### Culture media

Two media were used, CHOI4 medium [25] or CHOI5 medium, depending on experiments. CHOI5 medium is identical to CHOI4 medium except that it contained 33 wt% less ammonium chloride.

### Inoculum preparation

Starting from cultures maintained on CHOI4 plates at 4°C, 250-300 mL-shake flasks containing 50 mL of medium were inoculated and incubated at 30°C, 260 rpm, for approximately 48 h. The medium contained 0.2 vol% methanol as sole carbon source. Incubation was extended to 72-96 h in the case of the recombinant *M. extorquens *strains. The medium used to grow the recombinant strains also contained tetracycline at 15 mg/L.

### Cell cultivation for PHA production

Most experiments were conducted using 2 L-shake flasks containing 500 mL of either CHOI4 medium or CHOI5 medium. The media contained an initial concentration of 1 vol% methanol. Media used with the recombinant strains contained tetracycline at 15 mg/L. For induction of the recombinant *phaC *genes, cumate (4-isopropylbenzoic acid) was added to a final concentration of 20 mg/L. Whenever applicable, addition of the secondary substrate (carboxylic acids) was usually done after 48 or 72 h, or as specifically indicated in the Results and Discussion section. 1 M KOH was used for manual pH control.

Fermentation was performed in a 20 L-bioreactor (Chemap, Switzerland) that was inoculated with 10 vol% of a 72 h pre-culture grown in shake flasks. Upon oxygen depletion, methanol (usually 1 vol%) was manually added with a syringe. The co-substrate 7-octenoic acid (C8=) was supplied by the same means. Shortly prior to co-substrate addition, expression of the PHA synthase gene *phaC2 *was induced by adding 4-isopropylbenzoic acid to reach a final concentration of 20 mg/L. Microbial growth was monitored by spectrophotometry at 600 nm.

### Construction of the recombinant plasmids

**Figure 1:**
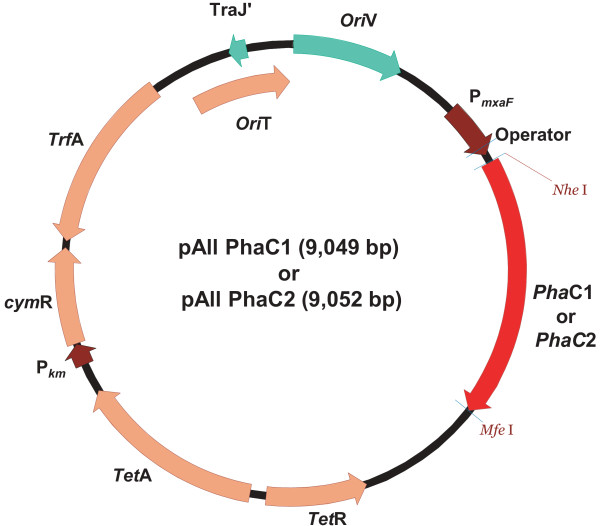
**Expression vector**. Genetic construction of recombinant plasmids containing the *P. fluorescens *GK13 polyhydroxyalkanoate (PHA) synthase genes *phaC1 *and *phaC2*, respectively.

To clone the two polyhydroxyalkanoic acid (PHA) synthase genes *phaC1 *and *phaC2 *from *Pseudomonas fluorescens *GK13, the genomic DNA of *P. fluorescens *GK13 was isolated and subjected to PCR using, for *phaC1*, the primers PhaC1FNhe (5' -CGC TAG CAT GAG CAA CAA GAA CAA TGA AGA CCT GCA GCG C- 3') (the *Nhe*I site is underlined), PhaC1RMFE (5' -GCA ATT GTC AAC GTT CGT GGA CAT AGG TCC CTG G- 3') (the *Mfe*I site is underlined) and, for *phaC2*, the primers PhaC2FNhe (5' -CGC TAG CAT GCG AGA GAA ACA GGT GTC GGG AGC CTT G- 3') (the *Nhe*I site is underlined), PhaC2RMFE (5' - GCA ATT GTC AGC GCA CGT GCA CGT AGG TGC CGG G- 3') (the *Mfe*I site is underlined), thus, yielding 1680-b*ρ *and 1683-b*ρ *PCR products, respectively. The PCR products were digested with *Nhe*I and *Mfe*l, and cloned into pAll-gfp (Choi *et al*., unpublished) digested with the same restriction enzymes to generate pAll-*phaC1 *and pAll-*phaC2*, respectively (Figure 1).

### Attempts to develop a *phaC*-minus *M. extorquens *strain *via *gene knock-out

Attempts were made to engineer a *phaC *-minus mutant of *M. extorquens via *gene knock-out of the native *phaC *gene using the TargeTron™Gene Knockout system from Sigma-Aldrich (Oakville, ON, Canada). A good number of mutants were obtained and PCR detection of the integrated intron in 9 randomly selected mutants showed the presence of the insert in all of them and its absence in the wildtype *M. extorquens *strain. However, all the mutants grew very poorly on methanol and, therefore, they could not be employed for further genetic engineering work.

### Electroporation of *M. extorquens*

Competent *M. extorquens *cells were prepared as previously described [28,29]. Competent cells were mixed in an Eppendorf tube with 1.0 g of recombinant plasmid DNA (pAll-*phaC1 *or pAll-*phaC2 *) and the tubes placed on ice for 20 min. The mixtures were transferred to an ice-cold electroporation cuvette and treated in a Bio-Rad electroporator (25 *μ*F, 200 Ω, 5 ms, 2.5 kV/cm). Immediately thereafter, 1 mL of CHOI medium [25] was added to the cuvette. The cell suspension was transferred to a 15 mL tube and incubated at 30°C for 5 h, then, 100 *μ*L of culture was spread on selective plates (CHOI agar with 35 *μ*g of tetracycline per mL). The plates were incubated at 30°C for 48 h until colonies appeared. Typically, about 300-500 transformants per plate were obtained.

### SDS-polyacrylamide gel electrophoresis

Crude extracts of recombinant *M. extorquens *cultures were prepared using a French press and protein concentration was estimated by the method of Bradford [30] using the Bio-Rad protein assay kit, with bovine serum albumin as standard. The cell extract samples were diluted in SDS-PAGE Loading Buffer and they were loaded at 10 *μ*g per well on a 4-12% NuPAGE Novex gel (Invitrogen Corp. Carlsbad, CA, USA). Gels were stained with Coomassie Brilliant Blue R-250.

### Extraction, purification and solvent fractioning of PHAs

Culture samples were centrifuged and the resulting pellets were washed in distilled water, methanol and, again, in distilled water followed by re-centrifugation after each washing step. Finally, the biomass was resuspended in water and freeze-dried (LyoStar, Model MNL-055-A, FTS Systems, Stone Ridge, NY). Lyophilized biomass was leached in approximately 20× volume (v/w) of chloroform overnight in a rotary shaker at 30°C. The soaked cells were then filtered through Whatman #4 paper (Maidstone, UK) and the filtrate purged with nitrogen. Resulting polymer gels were re-dissolved in chloroform and subjected to precipitation overnight in 10× volume (v/v) methanol at 4°C. Finally, precipitates were filtered through one sheet of Fisher Scientific P5 paper (Hampton, NH) and air-dried.

Freeze-dried biomass resulting from fermentation was leached in 20× volume (v/w) of chloroform or acetone for at least 6 h in a Branson model 5200 sonicator (Danbury, CT) at 60°C. The soaked cells were then vacuum-filtered through Whatman #4 paper (Maidstone, UK) and the filtrate boiled down in a Rotavapor (Büchi, Switzerland). Resulting polymer gels were re-dissolved in chloroform and subjected to precipitation overnight in 10× volume (v/v) methanol at 4°C. Finally, precipitates were vacuum-filtered through two sheets of Fisher Scientific P5 paper (Hampton, NH) and air-dried. Chloroform-extracted PHAs were fractioned by hot acetone in a Soxhlet apparatus as previously described [31].

### Analysis of the PHAs

#### GC-FID

GC-FID analysis was carried out as follows. Biomass samples were centrifuged in 50 mL conical Sarstedt tubes (Nümbrecht, Germany) and the pellets lyophilized. Methanolysis was used for analyzing the intracellular PHA content. Briefly, the dry biomass was treated with acidified methanol in the presence of benzoic acid as internal standard at 100°C for 3 h to convert 3-hydroxyalkanoate monomers to the corresponding methyl esters. The methyl esters were extracted in chloroform for subsequent analysis in a gas chromatographic system (Agilent 6890 GC-FID; Agilent Technologies, Wilmington, CA). PHBV from Sigma-Aldrich (Oakville, ON, Canada) and PHBHx from Procter & Gamble (Cincinnati, OH) were used as PHA standards. Purified biopolyesters were analyzed by the same method.

#### NMR

##### Equipment and general conditions used

Solution NMR spectra were acquired at 25°C on samples dissolved in deuterated chloroform on a Varian Inova 600 MHz spectrometer equipped with a HCN coldprobe and Z gradient. ^1^H 1D spectra were acquired with a sweep width of 12000 Hz and 128 transients. Assignment of ^1^H and ^13^C resonances were achieved *via *^1^H-^13^C correlated spectra detecting naturally abundant ^13^C. The ^1^H-^13^C Heteronuclear Single Quantun Coherence (HSQC) [32] and multiplicity edited HSQC spectra were acquired with 2048(t_2_) × 200(t_1_) complex points and ^1^H and ^13^C sweep widths of 10000 and 27145 Hz, respectively. Broadband ^13^C decoupling was achieved during acquisition using WURST2 decoupling over 140 ppm. Typically, 128 scans were acquired for each t_1 _value. HMBC (Heteronuclear Multiple Bond Correlation, [33]) and H2BC (Heteronuclear 2-Bond Correlation, [34]) experiments were acquired with 200 complex (H2BC) and real (HMBC) points in t1 with ^1^H and ^13^C sweep widths of 10000 and 36192 Hz, respectively and 256 transients. All 2D spectra were processed with nmrPipe [35] and analyzed using NMRView [36]. PPM values are quoted relative to TMS. A detailed description for an example of applied NMR analysis is provided as Appendix.

### Chemicals

Pentanoic acid (C5) was obtained from A & C American Chemicals Ltd. (Saint-Laurent, QC, Canada), 99% purity; 5-hexenoic acid (C6=) was obtained from either Sigma-Aldrich or TCI America (Portland, OR), both at 98% purity; 7-octenoic acid (C8=) was from Richman Chemical Inc. (Lower Gwynedd, PA), 98% purity. The following carboxylic acids were all from Sigma-Aldrich: 10-undecenoic acid (C11=), 98% purity; 4-pentenoic acid (C5=), 97% purity; *trans-2*-pentenoic acid (*t *- C5=), 98% purity; hexanoic acid (C6), 99% purity.

## Results and Discussion

### Production of unsaturated PHAs using the wild-type strain

The potential of the pink facultative methylotroph *M. extorquens *ATCC 55366 was tested to utilize various fatty acids including fatty acids with C-C double bonds. The assays were performed in shake flasks using medium CHOI4 or medium CHOI5. Nearly 100 shake flask assays were conducted. The *M. extorquens *cultures were grown first on methanol as sole carbon and energy source and the fatty acid of interest was added at some point to the growing cultures, generally at a final concentration in the 0.1-0.3% range. A summary of the substrates tested and of the results obtained is presented in Table 1. Only the homopolymer PHB or the copolymer PHBV was detected in the cultures as no other peaks apart from 3-hydroxybutyrate (3HB) and 3-hydroxypentanoate (3HP = 3HV) were seen in GC chromatograms. As can be seen, feeding of C5-fatty acids led to accumulation of the copolymer PHBV solely and, unfortunately, no trace of C-C double bounds could be detected in the PHAs produced upon feeding C5-fatty acids containing a C-C double bond. Control of pH is a key factor when cultures are fed free carboxylic acids. In the present study, efforts were made to minimize pH effects by regular, manual addition of 1 M KOH in order to maintain pH within a reasonable range favorable for growth and substrate utilization. Given the extent of our screening efforts, it may be reasonably concluded that the wildtype *M. extorquens *ATCC 55366 strain was unable to accumulate functionalized PHAs containing C-C double bonds. As a consequence, the next step consisted in developing *M. extorquens *strains harboring heterologous PHA synthase genes allowing for biosynthesis of the wanted functionalized PHAs.

**Table 1:** Utilization of various fatty acids, unsaturated or saturated, for growth and PHA accumulation by *M. extorquens *ATCC 55366.

Co-substrate	**Monomeric composition of the produced PHA**^**1**^	
	C4:0 [mol%]	C5:0 [mol%]
C5	30.4	69.6
C5=^2^	100	-
C5=	> 99	tr^3^
*t*-C5=	> 99	tr
*t*-C5=	87.9	12.1
C6	> 99	tr
C6	> 99	tr
C6	100	tr
C6=^4^	100	-
C8=	98.7	1.2
C8=^5^	100	-
C11=^6^	> 99	tr
C11=^7^	100	-

^1^CX:Y = double bond at position Y of monomeric chain length X;

^2^Three (3) similar samples were analyzed;

^3^tr = detected in trace quantity;

^4^Six (6) similar samples were analyzed;

^5^Three (3) similar samples were analyzed;

^6^Eight (8) similar samples were analyzed;

^7^Three (3) similar samples were analyzed.

### Metabolic engineering of *M. extorquens *for modifying its PHA-synthesizing machinery

The inability of the wildtype *M. extorquens *strain to yield the desired PHAs was assumed to be due to a too narrow substrate specificity of the wildtype PHA synthase enzyme. As done by several research groups [37-44], we then decided to engineer *M. extorquens *strains harboring different PHA synthase (*phaC*) genes known or assumed to code for PHA synthases of broader substrate specificity. After careful review of the literature, the *phaC1 *and *phaC2 *genes present in the *P. fluorescens *GK13 strain were selected because the PHA synthesis machinery of this bacterium is able to also produce longer PHA chains [23,45], an indication of broader substrate specificity. In a first step, the *phaC1 *and *phaC2 *genes were successfully isolated using PCR and the isolated genes were identical to the reported sequences [45]. In a second step, the two *phaC *genes were cloned into the pAll plasmid (Figure 1) and inducible expression was driven by the cloned methanol dehydrogenase (PmxaF) of *M. extorquens *under control of regulatory elements of *P. putida *F1. This new technology for inducible and regulated gene expression was developed by our group for *M. extorquens *[28,29]. It may be described as follows: (A) In the absence of the chemical inducer (p-isopropylbenzoate = cumate), the repressor protein cymR is bound to the operator site upstream of the gene of interest and transcription is blocked; (B) addition of cumate as inducer leads to formation of the cymR-cumate complex, followed by detachment of cymR from the operator and activation of the transcription of the downstream gene.

**Figure 2:**
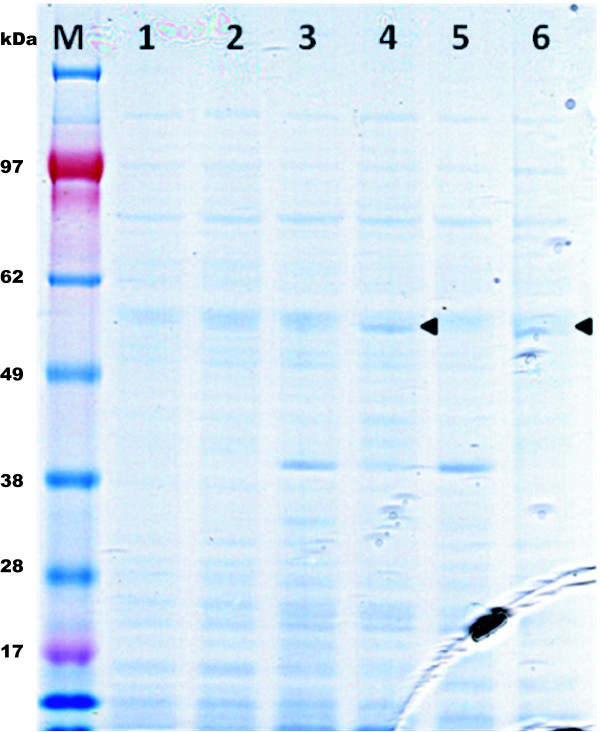
**SDS-PAGE (4-12%) of *M. extorquens *cell-free extracts from shake flask cultures**. Lane M, standard marker mixture; lane 1, wildtype *M. extorquens *before induction; lane 2, wildtype *M. extorquens *after induction with cumate; lane 3, *M. ex-pha**C1 ***before induction; lane 4, *M. ex-pha****C1 ***after induction; lane 5, *M. ex-pha****C2 ***before induction; lane 6, *M. ex-pha****C2 ***after induction. The arrows indicate the putative presence of recombinant PhaC1 or PhaC2 enzyme, which showed an approximate molecular mass of 62 kDa.

The two recombinant *M. extorquens *strains, *M. ex-pha****C1 ***and *M. ex-pha****C2***, as well as the *M. extorquens *wildtype strain were grown in CHOI medium containing methanol, plus tetracycline in the case of the two recombinant strains. As shown in Figure 2, addition of cumate, the inducer, led to expression of both the *phaC1 *gene (lane 4) and of the *phaC2 *gene (lane 6). Both protein bands indicated a molecular mass of approximately 62 kDa. Interestingly, a strong band, with an apparent molecular mass of 40 kDA, was present in the samples for noninduced *M. ex-pha**C1 ***cells (lane 3) or *M. ex-pha**C2 ***cells (lane 5). The same band was quasi inexistent in induced cells of the same recombinant strains. This additional band is certainly not related to the insert (*phaC *gene) since it has been observed with other recombinant *M. extorquens *strains harboring different and various inserts. Ideally, the two recombinant *phaC *genes should have been introduced into a *phaC *-minus mutant of *M. extorquens*. Unfortunately, although we were able to obtain such mutants, the mutants grew very poorly in the culture media containing methanol as sole carbon source. A similar observation on the growth of strain AMI of *M. extorquens *on C1 and C2 compounds was also reported by Korotkova and Lidstrom [46]. Consequently, because of the presence of the native PHA synthase, PHA blends comprised of PHB and functionalized PHAs could be expected.

### Growth of the two recombinant *M. extorquens *strains

**Figure 3:**
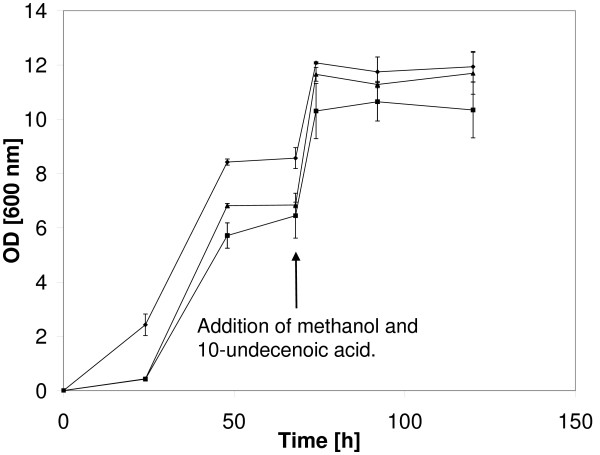
**Growth of *M. extorquens *strains**. Growth of the wildtype *M. extorquens *strain and of the two recombinant *M. extorquens *strains on methanol (Phase 1, 0-68 h) and on methanol + 10-undecenoic acid (C11=) (Phase 2, 68 h-end). Addition of the mixture is indicated by the arrow. (◆) wildtype strain, n = 3; (■) *M. ex-pha****C1 ***strain, n = 3; (▲) *M. ex-pha****C2 ***strain, n = 2. The medium initially contained 1 vol% methanol. At 68 h, methanol and C11= were added to give a final concentration of 2 vol% and 0.2 vol% of methanol and C11=, respectively.

As illustrated in Figure 3, the two recombinant strains grew well on methanol but maximal growth of the two strains was inferior to that of the wildtype strain. At 70-72 h, addition of a mixture of methanol + 10-undecenoic acid (C11=) resulted in further growth for the three strains but growth, as measured by optical density, rapidly reached a plateau in all three cases. The *M. ex-pha****C2 ***strain appeared to grow better than the *M. ex-pha****C1 ***strain under the conditions used. Phase contrast microscopy of the cultures showed the presence of granules, tentatively identified as PHB (or PHA) granules, in cells from the three *M. extorquens *strains and the granules increased in size over time. The appearance of the granules occurred between 68 h and 74 h and it coincided with the addition of the methanol + C11= mixture (Figure 3, arrow).

**Figure 4:**
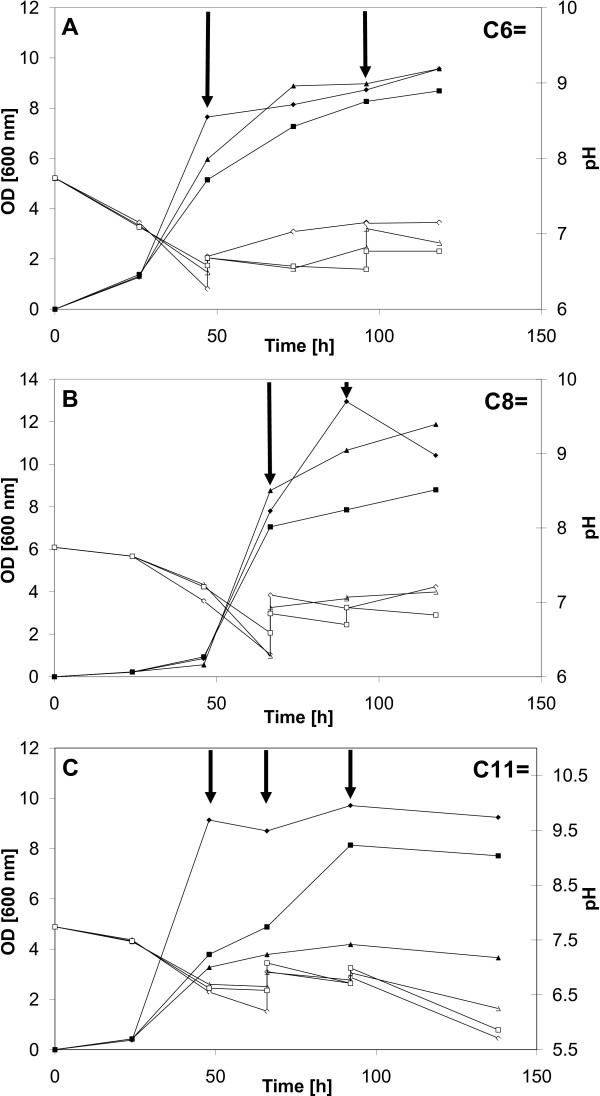
**Growth of the *M. ex-pha**C1 ***strain on selected unsaturated carboxylic acids**. Cultures were grown first on methanol, then, the selected unsaturated carboxylic acid was added pulse-wise (indicated by arrows). Partial control of pH was done using 1 M KOH to prevent intolerable extremes in pH. Closed symbols: OD at 600 nm, open symbols: pH. (A) 5-hexenoic acid (C6=); (B) 7-octenoic acid (C8=); (C) 10-undecenoic acid (C11=).

**Figure 5:**
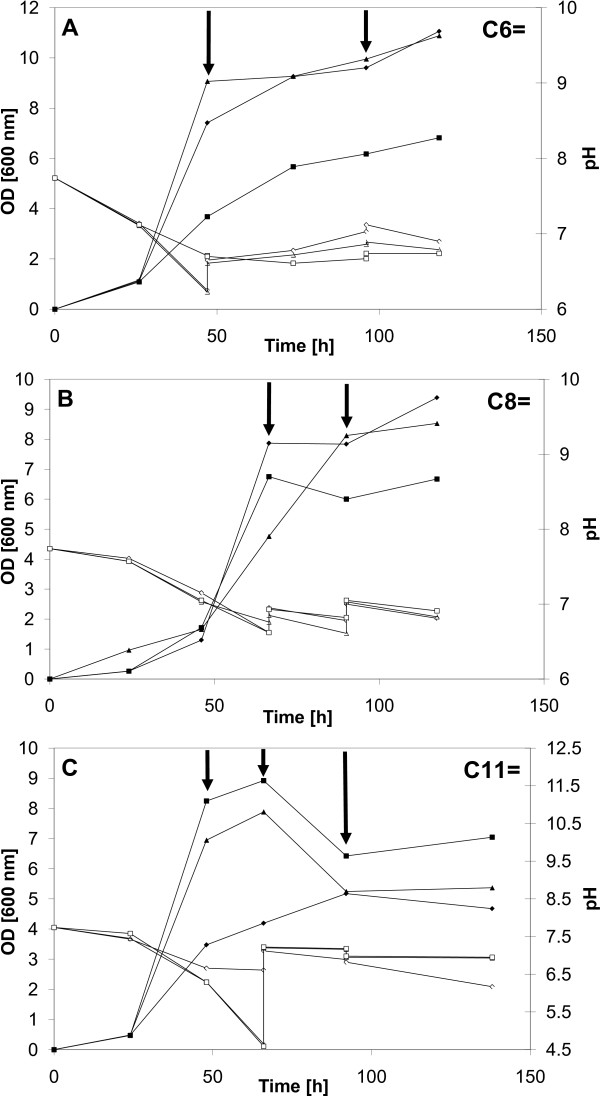
**Growth of the *M. ex-pha**C2 ***strain on selected unsaturated carboxylic acids**. Cultures were grown first on methanol, then, the selected unsaturated carboxylic acid was added pulse-wise (indicated by arrows). Partial control of pH was done using 1 M KOH to prevent intolerable extremes in pH. Closed symbols: OD at 600 nm, open symbols: pH. (A) 5-hexenoic acid (C6=); (B) 7-octenoic acid (C8=); (C) 10-undecenoic acid (C11=).

Many shake flask studies were conducted using various feeding strategies to verify the growth behavior in the presence of alkenoic acids. Cultures were started using methanol as main substrate and, at desired times, the selected unsaturated carboxylic acid was added to favor accumulation of a functionalized PHA. To minimize pH effects, attempts were made to maintain pH by manual addition of 1 M KOH. The growth results for strain *M. ex-pha**C1 ***are shown in Figure 4 while those for strain *M. expha**C2 ***are shown in Figure 5. Pulse addition of the carboxylic acid is indicated by arrows. The two *M. extorquens *strains (Figure 4A and Figure 5A) continued to grow, although more slowly, upon addition of 5-hexenoic acid (C6=) and "respectable" biomass levels (OD600nm) were obtained, between 8 and 11, with all flasks except for one case. The pH profile varied with each flask but pH values were always between 6.2 and 7.7. The growth results on 7-octenoic acid (C8=) are illustrated in Figure 4B (*M. ex-pha**C1***) and in Figure 5B (*M. ex-pha**C2***). The results were similar to those obtained with 5-hexenoic acid (C6=) except that the OD600nm profiles varied significantly more between flasks. As with 5-hexenoic acid, the pH profile varied with each flask but pH values were always between 6.2 and 7.6. With 10-undecenoic acid (C11=), however, growth appeared even more "erratic" as there was significant variation in the OD600nm profile between flasks (Figure 4C and Figure 5C for strains *M. expha**C1 ***and *M. ex-pha**C2***, respectively). Maximal OD600nm values varied between only 4 and almost 10 (Figure 4C). Overall, most of the biomass was obtained from methanol, *i.e.*, before addition of the respective carboxylic acid, due to the well-known toxicity of such acids. Probably due to the lack of acceptable pH control, significant differences in optical density profiles were observed between shake flasks, especially when C11= was fed (Figures 4C and 5C). Interestingly, in several cases, growth continued after addition of the "toxic" carboxylic acid. Several examples are depicted in Figures 4 and 5. The optical density values obtained are very comparable to values found in the recent literature for similar work [15,17,37-39,47]. In this study, as in many others, emphasis was on strain development, not on process optimization. With C11= as co-substrate, manual maintenance of the pH was also more problematic, pH values lower than 6 were recorded with both strains, reaching even near 4.5 at one point with strain *M. ex-pha**C2***.

### Determination of the monomeric composition of polyhydroxyalkanoates

Selected PHA samples were chosen as representatives and submitted to 1D and 2D NMR analysis for obtaining proof that C-C double bonds were present and that at least some of the C-C double bonds were located in the PHA side chains. Analysis of the NMR results confirmed both the presence of unsaturated PHA components in the samples and the presence of C-C double bonds in the side chains.

Consequently, the representative PHA samples, including a PHA sample produced by the wildtype *M. extorquens *strain, were submitted to 1D and 2D NMR analyses (see Materials and Methods) to further identify which unsaturated components might be present. The results are summarized in Table 2. The two samples derived from C11= yielded poor quality spectra. As representative example, the results for a PHA that was produced by *M. ex-phaC2 *on methanol and 7-octenoic acid will be presented in more detail:

**Table 2:** 1D and 2D NMR analysis of selected PHA samples^1^.

Co-substrate	1D Analysis	2D Analysis
C6=^2^	3HB as major peak (> 90%), 3HHx=^3^	Not performed.
C8=^4^	3HB as major peak (> 96%)	At least 5 minor components: 3HP, 3HHx, 3HHx=, 3HO and 3HO=.
C11=^5^	Poor quality spectra.	
-	3HB, 3HP (very minor)	No detectable unsaturated bonds

^1^These samples were chosen as representatives due to availability of enough material and equipment;

^2^Three (3) similar samples were analyzed;

^3^Since C6= was the co-substrate, it was concluded that the detected double bonds belonged to 3HHx=;

^4^Three (3) similar samples were analyzed;

^5^Two (2) similar samples were analyzed.

**Figure 6:**
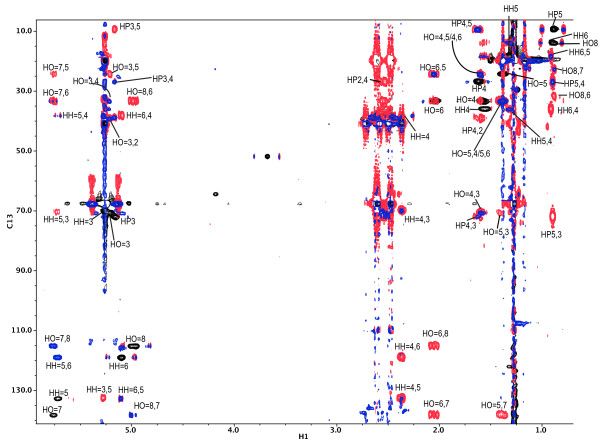
**Overlay of the 2D spectra for a PHA sample derived from methanol and 7-octenoic acid metabolism**. The 2D ^1^H-^13^C HSQC, HMBC and H2BC spectra are shown as black, red and blue contour plots, respectively. Single and multiple bond correlations for HP (3HP), HH (3HHx), HH= (3HHx=), HO (3HO) and HO= (3HO=) are indicated according to the following nomenclature. Multiple bond (HMBC) and 2-bond (H2BC) correlation cross peaks are identified by two numbers: the first corresponding to the ^1^H atom and the second relating to the ^13^C atom, e.g., HH6,4 describes the cross peak between H-6 and C-4 of 3-hydroxyhexanoate. Single correlation (HSQC) cross peaks are denoted by a single number (e.g., HP5, corresponds to the correlation of H-5 and C-5 for 3-hydroxypentanoate). Detailed analysis are provided as Appendix.

1. The ^1^H 1D spectrum indicated that 3HB was the major component comprising over 96% of the total signal (spectrum not shown). Using natural abundance 2D ^1^H-^13^C correlation spectra (Figure 6), it was possible to identify at least 5 minor components present which were assigned to 3HP, 3HHx, 3HO, 3HHx= and 3HO=. Chemical shift assignments for these components are shown in Table 3. Finally, at very low contour levels, we were able to detect and assign resonances at the terminus of polymer (these are too weak to detect in Figure 6 but assignments are listed in Table 3);

**Table 3:** Summary of the ^13^C-NMR shift values for a PHA sample derived from methanol and 7-octenoic acid metabolism.

Carbon	3HB	3HP	3HHx	3HHx=	3HO=	C-terminus
1	169.2	169.3	169.2^1^	169.2^1^	169.2^1^	-
2	40.7	38.9	39.3	39.5-40.8^1^	39.5-40.8^1^	43.2 (CH_2_)
3	67.7	71.9	70.7	70.06	70.8	64.5(CH)
4	19.7	26.9	35.9	38.2	33.3	22.5(CH_3_)
5	-	9.4	18.4	132.7	24.2	-
6	-	-	13.8	118.9	33.2	-
7	-	-	-	-	138.3	-
8	-	-	-	-	115.2	-

^1^Actual value is uncertain due to overlap from major component (3HB).

2. A notable feature of the 2D HSQC spectrum was the presence of high frequency ^1^H (5.0 ppm and 5.7 ppm) and ^13^C (115 ppm and 135 ppm) resonances indicative of double bonds. These were assigned to the 3HHx= and 3HO= monomers. Further analysis of the NMR observations indicated that the double bond for these two monomers must be at the end of the alkyl chain;

3. Using the C3 resonances in the HSQC, we obtained a ratio of 3HB:3HO= (1:0.06), 3HB:3HHx= (1:0.05), 3HB:3HP (1:0.02), 3HB:3HHx (1:0.008, comparing methyls), 3HB:3HO (1:0.008, comparing methyls).

Unfortunately, 2D NMR could not be performed with every sample due to cost and equipment availability. However for GC-FID analysis, it was now obvious that 3-hydroxyalkanoate monomers derived from 5-hexenoic acid would result in peaks for 3HHx= and 3HHx, while peaks for 3HP, 3HHx=, 3HHx, 3HO= and 3HO could be expected from metabolism with 7-octenoic acid. Consequently, GC-FID was routinely used thereafter to determine the composition of the PHAs produced and to look for the presence of C-C double bonds. Peaks for 3HB, 3HP and 3HHx were identified from corresponding methanolized PHA standards (see Materials and Methods). Since methyl esters resulting from 3-hydroxyalkenoates have a lower molecular weight compared to their saturated analogs, the peak that was recorded shortly prior to 3HHx was identified as 3HHx=. In addition to thereby related peaks for 3HB, 3HP, 3HHx= and 3HHx, GC chromatograms of PHA samples derived from methanol and 7-octenoic acid metabolism showed two more peaks that eluted at later time. From 2D NMR analysis it was concluded that these remaining peaks had to correspond to 3HO= and, eluted immediately thereafter, to 3HO. Peak areas were used to calculate the proportional monomeric compositions.

### Potential of the two recombinant strains for producing functionalized PHAs

From the start, our intention was to develop *M. extorquens *strains capable of accumulating functionalized PHAs harboring C-C double bonds within the side chains. As done by others, the production of such PHAs requires feeding an unsaturated carboxylic acid in the hope that a portion of the acid will be incorporated into the PHA molecule by the recombinant PhaC1 or PhaC2 enzyme, thus, leading to the potential presence of C-C double bonds in the PHA side chains.

Numerous shake flask studies were conducted using various unsaturated carboxylic acids, various feeding regimes and process conditions in the hope of identifying initial conditions that could lead to maximization of functionalized PHA production. Resulting PHA samples were generated and analyzed according to NMR and/or GC-FID analysis. As Table 4 indicates, the *M. ex-pha**C1 ***strain was able to produce PHA containing a small percentage, 0.75% +/- 0.57%, of C6:5 bond-containing material starting from C6=. The same strain, however, was very poor at incorporating C-C double bonds when either C8= or C11= was fed; only trace amounts of C-C double bonds were detected. Interestingly, the *M. ex-pha**C1 ***strain was able to incorporate significant quantities of a C5 unit starting from C8=. In this regard, C8= appeared much superior as chemical donor. Due to the high toxicity of the carboxylic acids used, cell densities were always low, generally between 1 and 2 g/L, on a dry weight basis.

**Table 4:** Chemical composition of the PHAs extracted from recombinant *M. extorquens *cells grown on methanol + various unsaturated carboxylic acids^1^.

		**Monomeric composition of the produced PHA**^**2**^							
Strain	Substrate [mol/L]		DCW [g/L]	C4:0 [mol%]	C5:0 [mol%]	C6:5 [mol%]	C6:0 [mol%]	C8:7 [mol%]	C8:0 [mol%]
*phaC1*	C6 = ^3^	16.82	1.59 +/- 0.23	98.11 +/- 0.89	-	0.75 +/- 0.57	1.14 +/- 0.34	-	-
	C8 = ^4^	6.61	1.65 +/- 0.31	97.35 +/- 1.69	2.65 +/- 1.68	tr5	tr	tr	tr
	C11 = ^6^	3.43	1.11 +/- 0.28	100	-	-	-	-	-
*phaC2*	C6 = ^3^	16.82	1.64 +/- 0.46	91.24 +/- 5.23	-	4.76 +/- 2.94	4.00 +/- 2.30	-	-
	C8 = ^4^	6.61	1.65 +/- 0.31	84.69 +/- 4.87	5.28 +/- 1.13	2.66 +/- 0.98	2.36 +/- 0.98	5.01 +/- 1.82	tr
	C11 = ^6^	3.43	0.91 +/- 0.07	100	-	-	-	-	-

^1^The PHA samples presented here were obtained from experiments shown in Figures 4 and 5;

^2^CX:Y = double bond at position Y of monomeric chain length X;

^3^Average +/- standard deviation, n = 3;

^4^Average +/- standard deviation, n = 3;

^5^tr = detected in trace quantity;

^6^Average +/- standard deviation, n = 2.

As illustrated in Table 4, the *M. ex-pha**C2 ***strain appeared better than the *M. ex-pha**C1 ***strain at incorporating C6:5 bonds into PHA (C-C double bond at position 5 of C6 monomer chain length). An average of 4.76% +/- 2.94% of the PHA was made up of C6:5 bond-containing units upon feeding of C6=. The same strain appeared also better at incorporating a C6 monomer from either C6= or C8=. When C8= was fed, four of the five samples showed the obvious presence of C8:7 bond-containing units. The concentrations of C6:5 material and those of a C6 monomer were also significantly higher. A C5 monomer was detected in the samples derived from C6= but it was much more prevalent when C8= was fed, an observation valid for both recombinant strains. Feeding of C11=, as with the first strain, did not lead to production of PHA containing significant percentages of C-C double bonds. Cell densities, again, were quite low. Table 4 indicates that PHA containing small quantities of C6:5 material could be obtained with both strains after feeding C6= but only the *M. ex- pha**C2 ***strain could generate both C6:5 material and C8:7 material when fed C8=.

The results listed in Table 4 show that PHA material containing modest concentrations of unsaturated C-C bonds could be obtained with the two strains. Three strong conclusions may be drawn from the results. (1) Strain *M. ex-pha**C2 ***was better at producing PHA material containing C-C double bonds; (2) the same strain was better at producing PHA material containing C-C bonds from C8=; (3) both strains gave very poor results with C11=. Several groups have observed or hypothesized that PhaC2 synthases have a lower substrate specificity than PhaC1 synthases [37,38,40]. Other groups have suggested that PhaC1 and PhaC2 synthases have different functions [43,44,48]. Contrary to observations made with other bacteria, our *M. extorquens *strains gave poor results with C11= and, therefore, only C6= or C8= proved to be adequate secondary substrates.

### Initial bioreactor studies: validation of shake flask results

Growth of recombinant *M. ex-phaC2 *and production of PHAs in shake flasks were successfully validated in a 20 L-bioreactor (14 L as working volume). A total of 11 mL of 7-octenoic acid was added throughout the fermentation in four individual additions starting at 29 h of running time. After 96 h the final dry cell weight (DCW) reached 5 g/L with a 37% PHA content on a dry cell weight basis (data not shown). Polymers were extracted from biomass with chloroform and subjected to routine structural analysis. The monomeric composition reflected the results from shake flask experiments when 7-octenoic acid was fed (Tables 4 and 5). As discussed previously, PHAs produced by recombinant *M. extorquens *strains with two different PHA synthases were most likely blends of homo- and copolymers. To test for this hypothesis, extracted polymers were soaked in acetone to possibly separate SCL-PHAs from MCL-3HA-containing PHAs. As opposed to MCL-PHAs, poly(3-hydroxybutyrate) (PHB) is only soluble in acetone when it is amorphous [49,50]. PHB separated from biomass, however, is crystalline and insoluble in acetone. As listed in Table 5, Soxhlet extraction was accompanied by a loss in PHA material. The remaining polymers were resolved into two different fractions by hot acetone. As expected, *M. ex-phaC2 *produced blends made up of SCL-PHAs and SCL/MCL-PHAs. Since the native PhaC enzyme is not capable of incorporating monomers other than SCL-3HA, the production of SCL/MCL-PHAs must have resulted from the action of the recombinant PhaC2 enzyme. Random abundance of the different monomers is likely the reason why some MCL-3HA units remained in the acetone-insoluble fraction. It is suggested here that the MCL-3HA content was very small in some copolymeric chains so that they could not dissolve in acetone.

**Table 5:** Solvent fractionation to separate SCL/MCL-PHAs from SCL-PHAs.

		**Monomeric composition of PHA**^**1**^					
PHA sample	**Portion**^**2 **^**[%]**	C4:0 [mol%]	C5:0 [mol%]	C6:5 [mol%]	C6:0 [mol%]	C8:7 [mol%]	C8:0 [mol%]
Blend^3^	-	91.36 +/- 1.18	0.83 +/- 0.02	3.26 +/- 1.00	1.31 +/- 0.05	3.24 +/- 0.11	tr4
Acetone(-)^5^	90.84	96.55	0.48	1.47	0.47	1.03	-
Acetone(+)^6^	4.63	55.85 +/- 6.43	3.33 +/- 0.70	21.72 +/- 7.81	6.98 +/- 1.56	12.12 +/- 3.48	tr
Loss^7^	4.52	-	-	-	-	-	-

^1^CX:Y = double bond at position Y of monomeric chain length X;

^2^Portion after Soxhlet fractionation;

^3^Chloroform extract from biomass (n = 2);

^4^tr = detected in trace quantity;

^5^Acetone-insoluble fraction (n = 1);

^6^Acetone-soluble fraction (n = 3);

^7^PHA loss during Soxhlet extraction.

Our experimental PHA samples from shake flask and fermentation experiments exhibited mainly SCL-3HA monomers (3HB, C4 and 3HP = 3HV, C5). Due to the presence of two unrelated *phaC *genes in our recombinant strains, we conclude that a major portion of the materials consisted of PHB or PHBV resulting from the native PHA synthase. Since the PHA synthase coded by the *phaC2 *gene of *P. fluorescens *GK13 was shown to have a broad substrate specificity [23], we conclude that the remaining biopolyesters of the polymer blends belonged to the highly valuable class of short-chain-length/mediumchain-length PHAs (SCL/MCL-PHAs, 4 ≤ C ≤ 14) that have been proposed for many applications due to their desirable thermo-mechanical properties [23]. For this reason, PHBHx with a 3HHx content of 20% and less is currently under investigation for its potential to function as tissue engineering material ([14] and reference therein). Based on our results, we have successfully added functionality to this highly regarded class of PHAs by incorporating MCL-3HA units bearing terminal double bonds.

## Conclusions

It was found that the wildtype *M. extorquens *ATCC 55366 strain could not produce functionalized PHAs starting from a methanol + unsaturated carboxylic acid mixture. As a consequence, a metabolic engineering approach was used to convert the wildtype strain into a "cell factory" capable of producing functionalized PHAs containing C-C double bonds. The presence of the C-C double bonds in the PHA side chains was confirmed by NMR. It was also found that the *M. extorquens *cell factory harboring the *phaC2 *gene appeared superior at utilizing unsaturated carboxylic acids and at incorporating C-C double bonds into PHA starting from either C6= or C8=. Our two *M. extorquens *cell factories were able to produce functionalized short-chain-length/mediumchain-length PHAs (SCL/MCL-PHAs), which combine desirable functionality of some MCL-PHAs with, reportedly, superior thermo-mechanical properties of inert SCL/MCL-PHAs. Double bonds may be used to induce side chain crosslinking and to covalently bind small or macromolecules upon chemical or enzymatic modifications. To date, the advantage of exhibiting functionality has been exclusively reserved for MCL- and LCL-PHAs.

## Abbreviations

3HA: 3-hydroxyalkanoate; 3HB (C4:0): 3-hydroxybutyrate; 3HHx (C6:0): 3-hydroxyhexanoate; 3HHx= (C6:5): 3-hydroxyhex-5-enonate; 3HO (C8:0): 3-hydroxyoctanoate; 3HO= (C8:7): 3-hydroxyoct-7-enoate; 3HP (C5:0): 3-hydroxypentanoate; 3HV (C5:0): 3-hydroxyvalerate; C5: pentanoic acid; C5=: 4-pentenoic acid; *t*-C5=: *trans*-2-pentenoic acid; C6: hexanoic acid; C6=: 5-hexenoic acid; C8:= 7-octenoic acid; C11=: 10-undecenoic acid; CX:Y: double bond at position Y of monomeric chain length X; DCW: dry cell weight; GC-FID: gas chromatography-flame ionization detector; H2BC: Heteronuclear 2-Bond Correlation; HMBC: Heteronuclear Multiple Bond Correlation; HMQC: Heteronuclear Multiple Quantum Coherence; HSQC: Heteronuclear Single Quantum Coherence; KOH: potassium hydroxide; LCL: long-chain-length; MCL: medium-chain-length; MeOH: methanol; NMR: Nuclear Magnetic Resonance spectroscopy; OD: optical density; PHB: poly(3-hydroxybutyrate); PHA: polyhydroxyalkanaote; PHBV: poly(3-hydroxbutyrate-*co*-3-hydroxyvalerate); PHBHx: poly(3-hydroxybutyrate-*co*-3-hydroxyhexanoate); PPM: parts per million; SCL: short-chain-length; TMS: tetramethylsilane.

## Competing interests

The authors declare that they have no competing interests.

## Authors' contributions

PH as PhD student carried out most of the experimental work and co-wrote the manuscript. PH also selected the two PHA synthase genes that were cloned into *M. extorquens*, performed the initial bioreactor studies, participated into some of the molecular biology work and analyzed all results from GC-FID. YJC and CBM used an emerging expression cassette developed by themselves and colleagues for cloning the two PHA synthase genes and for delivering a number of recombinant strains. MO performed the NMR work and was responsible for data analysis thereof. PV as PhD director and DG as co-director coordinated the study and offered general and expert supervision. All authors read and approved the final manuscript.

## Appendix

### Detailed analysis of NMR results

Selected PHA samples were submitted to 1D and 2D NMR analyses to further identify which unsaturated components might be present. As example, the treatments for a PHA that was produced from methanol and 7-octenoic acid will be presented in detail. The ^1^H 1D spectrum indicated that 3HB was the major component comprising over 96% of the total signal (spectrum not shown). Using natural abundance 2D ^1^H-^13^C correlation spectra (Figure 6), it was possible to identify at least 5 minor components present which were assigned to 3HP,3HHx, 3HO, 3HHx= and 3HO=. Chemical shift assignments for these components, based on the^1^H-^13^C Heteronuclear Single Quantum Coherence(HSQC), Heteronuclear Multiple Quantum Coherence (HMQC) and Heteronuclear 2-Bond Correlation(H2BC) 2D spectra (shown in Figure 6 as black, red and blue contour plots, respectively), are shown in Table 3. The 2D spectra indicated that 5 methyl groups were present. In addition to 3HB, the other methyl resonances were assigned to 3HP, 3HHx and 3HO, and the terminal methyl. All expected single double and multiple bond correlations were observed for 3HP and 3HHx. Further confirmation for 3HP was observed by the multiple, 3-bond correlation in the HMBC from the methyl protons (H5)to the C3 methine ^13^C chemical shift at 71.9 ppm. Two other C methyl resonances at 13.8 ppm, were alleviated by their ^1^H shifts at 0.91 and 0.88 ppm. The slightly higher frequency peak was assigned to3HHx and can be traced to the distinct C3 ^13^C chemical shift *via *the HMBC/H2BC spectra (Figure 6). Based on the ^13^C shifts for the H2BC (22.6 ppm) and HMBC (31.8 ppm) cross-peaks and comparison with previously published data [51,52], the slightly lower frequency ^1^H methyl resonance originated from a longer chained 3-hydroxyalkanoate. We have assigned this to 3HO (based on the fact that we observed the related 3HO= polymer, see below) although we cannot exclusively rule out other longer chained monomers. Finally, at very low contour levels, we were able to detect and assign resonances at the terminus of polymer (these are too weak to detect in Figure 6 but assignments are listed in Table 3).

A notable feature of the 2D HSQC spectrum was the presence of high frequency ^1^H (5.0 ppm and 5.7 ppm) and ^13^C (115 ppm and 135 ppm) resonances indicative of double bonds. These were assigned to the 3HHx= and 3HO= monomers based on the following reasoning. Firstly, the sign of these resonances in the multiplicity-edited HSQC (data not shown)showed the higher frequency ^1^H peaks (5.7 ppm) tobe CH resonances (C5, 3HHx= and C7, 3HO=) and the peaks at 5.0 ppm to arise from CH groups (C6 and C8 for 3HHx= and 3HO=, respectively). The H2BC spectrum (blue, Figure 6) showed a clear 2-bond correlation between, for example, the H of C8 and C7 of 3HO= (marked HO = 8,7 on Figure 6) and the H of C6 and C5 of 3HHx= (marked HH = 6,5 on Figure 6). Thus, the double bond for both monomers must be at the end of the alkyl chain. The 3HHx = monomer was assigned based on the characteristic chemical shift value for C3 in 3-hydroxyalkanoatemonomers of 70.5, and the observation of a 3-bond HMBC correlation between the ^1^H of C5 and a ^13^C peak at 70.06, which must be C3 (labeled HH = 5,3 on Figure 6). This correlation was confirmed by the observation of the remaining expected multiple and single bond correlations for 3HHx= which are shown on Figure 6. In contrast to 3HHx=, in 3HO=, the =CH resonance (C7 in 3HO=) showed a 3-bond HMBC correlation to a ^13^C peak at 24.2 ppm (labeled HO = 7,5 in Figure 6) which must belong to a CH (confirmed by the multiplicity edited HSQC experiment), indicating the alkyl chain is longer for this monomer. Assignment to 3HO= was once again based on the characteristic chemical shift of C3 andH3 at 70.5 ppm and 5.2 ppm, respectively. Notably, the C5 resonance just assigned showed a 3-bond correlation to a ^1^H resonance at 5.18 ppm, which is probably H3. This was confirmed by the observation of the H5 resonance exhibiting a 3-bond correlation in the HMBC to 70.9 ppm, which must belong to C3. Further analysis of the HMBC and H2BC spectra confirmed this assignment, showing almost all the expected single and double bond correlations. Using the C3 resonances in the HSQC we obtained a ratio of 3HB:3HO= (1:0.06), 3HB:3HHx= (1:0.05), 3HB:3HP (1:0.02), 3HB:3HHx (1:0.008, comparing10methyls), 3HB:3HO (1:0.008, comparing methyls).
